# Depth-Dependent Differences in Community Structure of the Human Colonic Microbiota in Health

**DOI:** 10.1371/journal.pone.0078835

**Published:** 2013-11-06

**Authors:** Aonghus Lavelle, Grainne Lennon, Neil Docherty, Aine Balfe, Hugh E. Mulcahy, Glen Doherty, Diarmuid O′Donoghue, John M. Hyland, Fergus Shanahan, Kieran Sheahan, J. Calvin Coffey, Desmond C. Winter, P. Ronan O′Connell

**Affiliations:** 1 University College Dublin, School of Medicine and Medical Science, Dublin, Ireland; 2 Centre for Colorectal Disease, St Vincent's University Hospital, Dublin, Ireland; 3 Department of Physiology, Trinity College Dublin, Ireland; 4 4i Centre for Interventions in Inflammation, Infection and Immunity, Graduate Entry Medical School, University of Limerick, Limerick, Ireland; 5 Alimentary Pharmabiotic Centre, University College Cork, Cork, Ireland; Loyola University Medical Center, United States of America

## Abstract

**Objective:**

The aims of this study were to develop techniques for spatial microbial assessment in humans and to establish colonic luminal and mucosal spatial ecology, encompassing longitudinal and cross-sectional axes.

**Design:**

A microbiological protected specimen brush was used in conjunction with a biopsy forceps to sample the colon in nine healthy volunteers undergoing colonoscopy. Terminal Restriction Fragment Length Polymorphism analysis was used to determine the major variables in the spatial organization of the colonic microbiota.

**Results:**

Protected Specimen Brush sampling retrieved region-specific, uncontaminated samples that were enriched for bacterial DNA and depleted in human DNA when compared to biopsy samples. Terminal Restriction Fragment Length Polymorphism analysis revealed a segmentation of bacterial communities between the luminal brush and biopsy-associated ecological niches with little variability across the longitudinal axis of the colon and reduced diversity in brush samples.

**Conclusion:**

These results support the concept of a microbiota with little longitudinal variability but with some degree of segregation between luminal and mucosal communities.

## Introduction

Humans are now recognized as a composite, co-evolved organism, with a large, co-evolved microbiota permitting mutualistic interactions [1], [2]. The role of the colonic microbiota in energy harvesting [3] and gut and immune maturation [4], [5], are topics of intense scientific investigation. Conversely, the potential role of the microbiota in the etiology of conditions such as allergy and asthma [6], inflammatory bowel disease [7], colorectal cancer [8], irritable bowel syndrome [9], obesity/metabolic syndrome [10] and even regulation of the gut brain axis [11], is highly topical, while a close link between the microbiota and diet and the progression of senescence has been recently established [12]. Identification of disease-specific microbial patterns may enable prophylactic and therapeutic manipulation of the gut microbiota [13]. While metagenomics, metatranscriptomics, metaproteomics and metabolomics are a rich source of insight into the microbiota, there is a need to integrate the spatial component to gut ecology.

Seminal papers published recently by the major microbiome consortia, the Human Microbiome Project and the MetaHIT project, have shed light on the nature of the microbiome by harnessing high-throughput sequencing techniques on a massive scale [14], [15]. These studies have used fecal samples as a starting material, allowing broad sampling of each individual's microbial gene complement, while conceding that there may be microbial niche-specific variability along the long- and cross-sectional axes of the colon [14]–[17]. Fecal sampling is readily adaptable to large patient cohorts, providing high yields of microbial DNA and permitting the assessment of temporal responses to environmental changes.

However, feces do not reflect microbial ecology at the epithelial interface [17]–[22]. Current information regarding variation of the microbiota along the longitudinal axis of the colon reveals a broadly homogenous pattern within individuals, with prominent inter-individual variability and notably, some degree of micro-heterogeneity between adjacent mucosal biopsies [20], [23]. Structurally, this is associated with an outer, colonized mucus gel layer separated from the epithelium by a dense layer of non-colonized mucus [24].

Attempts to categorize individuals based on analysis of stool microbiota alone have raised the possibility that with so much data and such a large degree of variability between individuals, it may be difficult to distinguish signal from noise, without first incorporating the other dimensions of microbial ecology [25]–[27]. While a core microbiota may prove elusive, reproducible patterns in the spatial structure of the microbiota, from the gross anatomical level down to fine-grained, ultra-structural interactions at the host–microbial interface, may provide insights that are difficult for large sequencing projects to discern. With regard to luminal contents, there are changes from the caecum to the rectum in terms of carbohydrate concentration, stool consistency, water content and pH, with pH falling in the cecum from values found in the distal ileum and then slowing rising across the colon to the rectum [28]. It is possible that such changes in colonic physiology might be reflected in the patterns of diseases such as ulcerative colitis and colorectal cancer that affect the colon in stereotyped, asymmetrical distributions, and may also be apparent in the microbiota [29].

In health, interaction between the microbiota and host immune system results in a state of controlled inflammation, while in disease states, consequent alterations to the microbiome may result in a vicious cycle, which perpetuates the underlying condition. Recently it has been shown that the microbiota can both transfer and mitigate metabolic syndromes [30], [31]. Understanding how this multidimensional, interlinked process occurs requires the integration of tools from spatial ecology with molecular microbiological methods to define temporal-spatial patterns of colonization in the gut.

**Figure 1:**
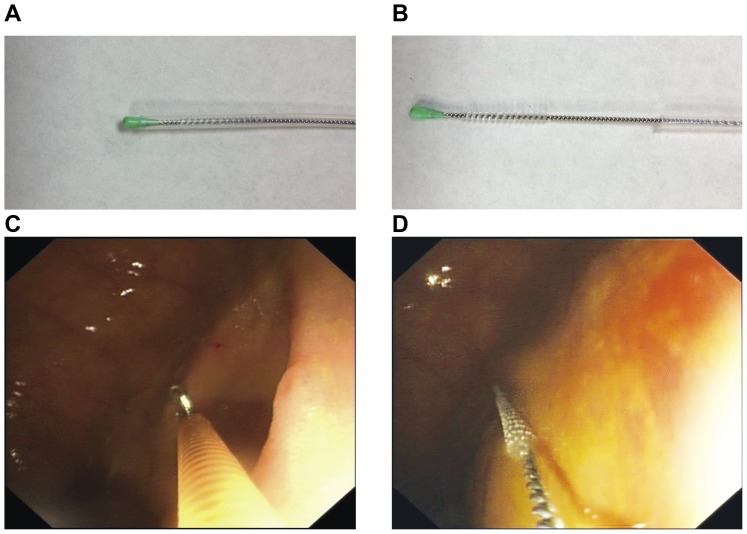
Protected Specimen Brush and biopsy sampling at endoscopy. A. The Hobbs medical protected specimen brush (PSB) in the closed position, with plug and sheath protecting the sampling brush from contamination within the colonoscope working channel. B. The PSB in the deployed position. C. A mucosal biopsy being retrieved at colonoscopy. D. The PSB deployed under direct vision at colonoscopy.

The first aim of this study was to develop and validate techniques for reproducible assessment of spatial variability in the colonic microbiota, combining conventional mucosal biopsy with microbiological protected specimen brushing (PSB) (Figure 1A and 1B), which employs a plug and sheath to protect the sample within the colonoscope working channel. The second aim was to apply these techniques to determine the major axial determinants of microbial biogeography in the colon, while validating a platform for the programmed assessment of disturbances in spatial ecology in colonic disease. The dual use of the colonoscope working channel for both instrumentation and suctioning of stool necessitated this approach to protect the samples. Additionally, by retrieving adjacent mucosal biopsies and luminal brushings (Figure 1C and 1D) from both the cecum and the rectum, we could determine whether the associated bacterial communities clustered predominantly by the colonic location from which they were sampled, or by their disposition with respect to the host-bacterial interface.

## Materials and Methods

### Ethics Statement

Ethical approval was obtained from St. Vincent's University Hospital Ethics and Medical Research Committee. All individuals gave informed, written consent prior to the procedure.

### Patient Recruitment

Nine healthy volunteers were recruited who were undergoing routine day case colonoscopy (Table 1) and were found to have no mucosal evidence of active pathology. Participants were greater than 18 years of age and had not taken antibiotics in the previous 3 months. Patients with a history of inflammatory bowel disease, colon cancer, colonic resection, active GI bleeding or hospital admission in the preceding six weeks, were excluded. Bowel preparations used were polyethylene glycol and sodium picosulphate based.

**Table 1:** Characteristics of study volunteers.

Characteristics	Subcategories	Results
Age (years)	Mean	48.2
	Range	25–71
Sex	Male	5
	Female	4
Bowel preparation	Picolax	5
	Kleanprep	4
Indication	PR bleeding	2
	Polyp surveillance	2
	PR discharge	2
	Fecal incontinence	1
	Family history cancer	1
	Abdominal pain	1

### Sample Collection

In the cecum, mucosal biopsies were taken using a Radial Jaw® 3 biopsy forceps (Boston Scientific, Natick, MA, U.S.A.) (Figure 1A). Mucosal biopsies sample the mucus gel layer, epithelium and variable amounts of the submucosa. The samples were retrieved with a sterile tweezers preventing fecal contamination from the outside of the forceps. The sample was immediately placed in a sterile, nuclease-free 1.5 ml microcentrifuge tubes (Greiner, Sigma-Aldrich, Baden-Württemberg, Germany) container and frozen at -20°C on dry-ice for storage at −80°C until DNA extraction.

Brush sampling was conducted with a Microbiological Protected Specimen Brush (Hobbs Medical Inc., Stafford Springs, Connecticut, USA). This brush targeted the superficial mucus gel layer from the luminal aspect, was deployed over glistening mucosa and avoided pools of fluid. This is a sterile, single-use, sheathed brush with a distal plug at the tip that seals the brush within the sheath during introduction and retraction through the colonoscope channel (Figure 1). The brush was deployed under direct vision. The brush was then sealed into the sheath and retracted as one. This was repeated in the rectum.

### DNA extraction

For brush samples, the plug and the tip of the wire were dissociated using sterile wire cutters, then placed in a sterile, nuclease-free 1.5 ml microcentrifuge tubes (Greiner, Sigma-Aldrich, Baden-Württemberg, Germany). DNA was extracted using a Qiagen DNA mini kit (Qiagen, Germany). Briefly, 180 µl of tissue lysis buffer (ATL buffer) was added along with 20 µl of proteinase K to each micro-centrifuge tube containing the sampling brush. This was vortexed vigorously for 1 minute to dislodge adherent mucus, followed by pulse centrifugation at 8,000 rpm for 5 seconds and incubation at 56°C for 1 hour. The tubes were intermittently removed from the heat-block during incubation and vortexed again to aid in bacterial cell wall lysis.

Following brush removal, 200 µl of a guanidine-based lysis buffer (AL) was added, pulse vortexed and incubated at 70°C for 10 minutes. Finally, 100 µl of 100% molecular grade ethanol was added and the mixture loaded onto Qiagen columns (Qiagen, Germany) and processed as per manufacturer's instructions. The final eluate of DNA was in 200 µl of elution buffer.

Biopsy samples were processed in a similar manner using a Qiagen DNA mini kit. Briefly, samples were cut using a sterile blade and vigorously vortexed in 180 µl of buffer ATL and 20 µl of proteinase K to maximize cell lysis and processed as described above.

### Conventional PCR

**Figure 2:**
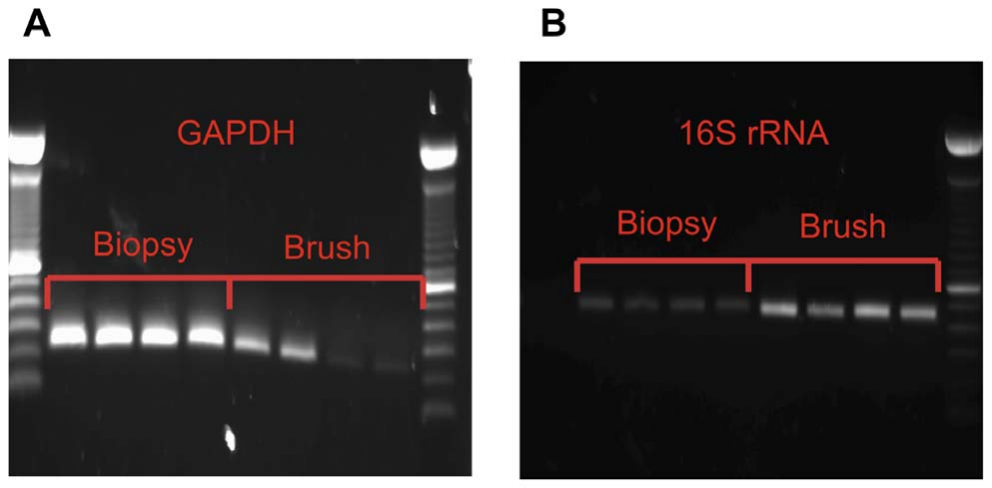
Brush sampling retrieves an enriched bacterial sample with less host eukaryotic DNA. A. Agarose gel with bands representing human GAPDH gene, illustrating the reduced quantity of human DNA in brush samples compared to biopsy samples. B. Agarose gel with bands representing the 16S rRNA pan-bacterial gene, illustrating the increased proportion bacterial DNA which is sampled by brushing compared to biopsy sampling.

Conventional PCR analysis targeted the human glyceraldehyde-3-phosphate dehydrogenase (GAPDH) gene (forward primer 5′-TGATGACATCAAGAAGGTGGTGAAG-3′ reverse primer 5′-TCCTTGGAGGCCATGTGGGCCAT-3′) and the 16S rRNA gene (forward primer 5′-TCCTACGGGAGGCAGCAGT-3′, reverse primer 5′-GGACTACCAGGGATCT AATCCTGTT-3′) (Eurofins MWG) (Figure 2). All PCR reactions were carried out using Go Taq^®^ Polymerase mix (Promega, Madison, WI, USA), on a Multigene thermocycler (Labnet, Woodbridge, NJ, USA), under the following thermocycling conditions: 95°C for 2 minutes followed by 35 cycles of 95°C, 62°C and 72°C, each for 30 seconds followed by a final extension at 72°C for 10 minutes. PCR products were analyzed by electrophoresis in a 1.5% agarose gel at 100 V for 60 min, followed by visualization under UV light. Positive, negative, and extraction controls were included for each PCR reaction.

### Quantitative real-time PCR analysis (qPCR)

qPCR analysis was performed in duplicate wells for each sample. Positive and negative controls were included for each PCR assay run. Real-time PCR was performed on an ABI 7900HT sequence detector (Applied Biosystems, Foster city, CA, USA) using universal thermal cycling conditions. ABI PRISM® Sequence detection system version 2.1 (Applied Biosystems) was used for all data analysis. A typical 20-µl real-time PCR amplification reaction contained 1X TaqMan Universal Mastermix (Applied Biosystems), the appropriate forward and reverse primers and MGBNFQ probe at concentrations of 300 nM and 175 nM respectively, and 4 µl of DNA extract adjusted to 1 ng/µl. The primer and probe set had been previously published [32], [33]. Each assay run incorporated a reference sample of cloned 16S rRNA gene from *Desulfovibrio desulfuricans* (ATCC 27774) bacteria [34]. Post PCR analysis involved determination of the pan bacterial copy number in each sample based on its fold change relative to a plasmid DNA standard. Subsequently, the calculated copy numbers were normalised for extract volume, and concentration. A plasmid DNA standards was generated for the purpose of determining the Pan Bacterial Copy number within each sample. This standard was included in each qRT-PCR assay run and used in a 2^−ΔCt^ calculation to determine the fold difference in Pan Bacterial Copy number within each sample. Briefly, *Desulfovibrio desulfuricans* (ATCC 27774) was cultured under anaerobic conditions in Postgate's medium and DNA was extracted using DNeasy® Tissue Kit (Qiagen, Hilden, Germany). A 466bp amplicon of the 16S rRNA gene, generated from the culture DNA extract as described previously (Rowen et al 2010) and cloned into pCR2.1-TOPO vector using the TOPO TA cloning system (Invitrogen, Groningen, The Netherlands) according to the manufacturer's instructions. DNA from the recombinant plasmid mini-preps was purified using the QIAprep® Spin Miniprep Kit (Qiagen). Total weight per recombinant plasmid was calculated and this was used to generate a series of DNA standards of known copy number of the target sequence.

### Terminal restriction fragment length polymorphism (T-RFLP)

**Figure 3:**
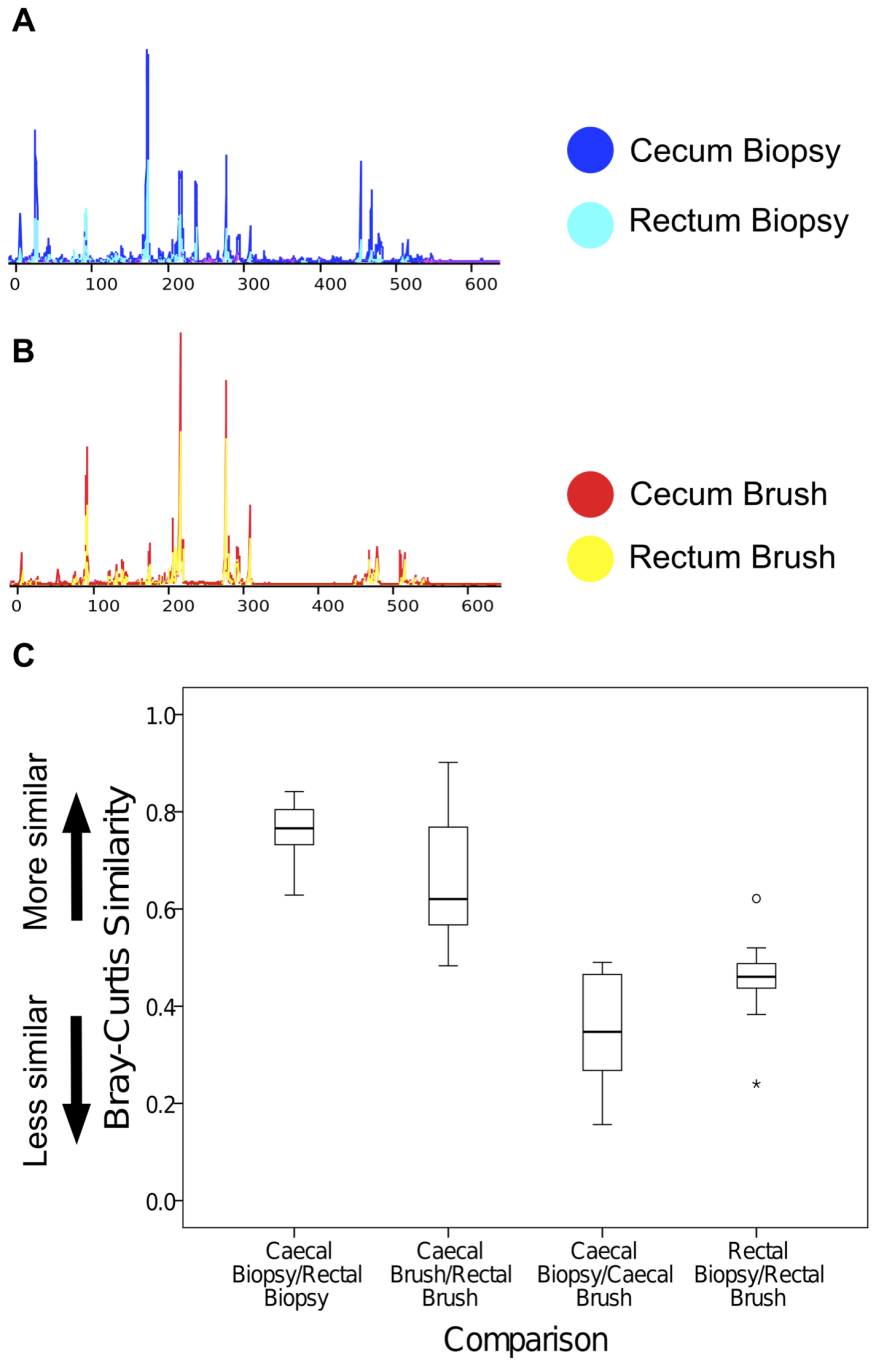
T-RFLP shows greater similarity based on sampling technique than colonic region within individuals. A. Electropherograms of T-RFLP sequence profiles generated from cecal and rectal biopsies from sample 1, superimposed on each other, illustrating close approximation of sample profiles. B. Electropherograms of T-RFLP sequence profiles generated from cecal and rectal brushings from sample 1. C. Mean Bray-Curtis Similarity Index (BCI) values between cecal and rectal biopsy samples (0.77 (0.73–0.81)), cecal and rectal brush samples (0.62 (0.57–0.77)) and biopsy and brush samples at cecum (0.35 (0.27–0.47)) and rectum (0.46 (0.44–0.49)).

T-RFLP analysis was applied to identify differences in bacterial ecology between (1) cecal and rectal regions of the colon and (2) brush and biopsy samples. T-RFLP amplifies a conserved region of the bacterial 16S rRNA gene with a fluorescently labeled primer then digests amplified product with a restriction enzyme. Fragment digests of differing lengths are generated and reflect species level differences in the 16S rRNA gene coding sequence. Fragment lengths are outputted in the form of an electropherogram and the incidence and relative abundance of fragments can be used as a proxy for species quantity and diversity (Figure 3A and 3B). Prior to analysis both spatial and spectral calibration of the data collection software was performed. Spatial calibration was performed to ensure alignment and optimal detection between capillaries. Criteria for evaluation included a single sharp peak for each capillary, reproducible peak heights, and spacing of 15+/−2 pixels between peaks. Spectral calibration was performed with the DS-33 Matrix Standard Kit (Dye Set G5) kit (Applied Biosystems) to correct for the overlapping of fluorescence emission spectra of the dyes. The threshold for the Q value (a measure of the consistency between the final matrix and the data from which it was computed) and C value (the upper and lower measure of the overlap between the dye peaks in the fluorescence emission spectra) were set to 0.9 and 4−7 respectively.

Following DNA purification the 16S rRNA gene was amplified by conventional PCR using a 6-carboxyfluorescein (6-FAM) labeled forward primer (6-FAM-8F AGAGTTTGATCCTGGCTCAG Integrated DNA technologies, Coralville, IA, USA) and a conventional reverse primer (AGAAAGGAGGTGATCCAGCC). PCR was performed using 70 ng of template DNA, 1x Go Taq® Polymerase mix (Promega, Madison, WI, USA) and 350 nM of primer mix in 50 µl reaction volumes. PCR cycling conditions were as follows: 94°C for 2 min followed by 45 cycles of 94°C for 30 sec/58°C for 45 sec/72°C for 90 sec. This was followed by a final incubation of 72°C for 4 min on a Multigene thermocycler (Labnet, Woodbridge, NJ, USA).

PCR amplicons were column purified using a QIAquick PCR purification kit (Qiagen, Germany) according to the manufacturer's protocol. 300 ng of the purified DNA was digested with 20 U of MspI restriction enzyme (Promega, Madison, WI, USA) for 2 hours at 37°C, and subsequently column purified using a QIAquick PCR purification kit (Qiagen, Germany) according to the manufacturer's protocol. The restricted products were eluted in a final volume of 30 µl of buffer EB.

Fragments were separated by capillary electrophoresis on a 3100 genetic analyzer (Applied Biosystems, Foster city, CA, USA) in GeneScan^TM^ mode as follows: 100 ng of the purified MspI restricted products were analyzed in duplicate on a CE plate to which a GeneScan^TM^ Liz 1200^®^ size standard (Applied Biosystems, Foster city, CA, USA) had been added. A control of 100 ng of undigested PCR product was run in duplicate on the same plate. The fluorescently labeled terminal fragments, which generated electropherogram peaks, were identified using Peak Scanner^TM^ Software v1.0 (Applied Biosystems, Foster city, CA, USA). Peaks corresponding to fragments of between 50 and 1000 base pairs in length were used for analysis. T-RFLP data is available upon request from the corresponding author.

### Statistical Analysis

Terminal restriction fragment (TRF) sizes were used in downstream analysis as a proxy for species presence and peak heights as a proxy for species abundance. Peaks present in both technical replicates were incorporated into a consensus profile of normalized, binned peaks using the software program T-align [35].

Bray-Curtis similarity indices were calculated using the vegan package in R and the results used to generate a dissimilarity matrix, which was imported into MEGA5 to create neighbor-joining dendrograms [36]–[38]. Shannon Diversity Indices (H′) and species evenness (E) were calculated separately in Microsoft Excel™ using the formulae: 
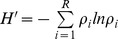
 and 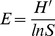 respectively, where ρ is the proportion of a TRF peak to total TRF abundance and S is the number of peaks. Graphing and statistical analyses were carried out in SPSS® v18.0 (IBM). Chromatogram visualization was performed using PeakStudio [39], while the vegan and rgl packages in R were used to perform and visualize non-metric multidimensional scaling (NMDS) and Analysis of Similarity (ANOSIM) [40]. The Mann-Whitney test was used to test for statistical significance and results of Bray-Curtis Index and Diversity measures are presented as medians with accompanying inter-quartile range (IQR), while DNA quantities and qPCR results are presented as mean and standard deviations.

## Results

Despite DNA yields, as measured by spectrophotometry, being lower in brush than biopsy samples (biopsy samples (6395 ng (SD 4232 ng)), brush samples (2636 ng (SD 1932 ng) (*P* = 0.024)), quantitative Real-Time PCR confirmed that the yield of bacterial DNA in the sample extracts was consistently higher in brush than biopsy samples (mean 4.075×10^9^ gene copies per sample (SD 2.77×10^9^) versus 1.45×10^8^ (SD 2.35×10^8^) 16S rRNA copies per sample, respectively *P*<.001). Conventional PCR and gel analysis provided visual confirmation that the proportion of human DNA in the biopsy samples was greater than that in brush samples, while the proportion of bacterial DNA was correspondingly higher in the brush samples than the biopsies (Figure 2A, 2B).

### Terminal restriction fragment length polymorphism analysis

The Bray-Curtis Index is a quantitative measure of shared species ranging from 0 (no shared species) to 1 (identical). This reflects the degree of similarity in microbial ecology. The median intra-patient pairwise Bray-Curtis similarity for biopsies was 0.77 (IQR 0.73–0.81) and that for brushes was 0.62 (0.57–0.77) (Figure 3). Thus cecal and rectal biopsies had a median bacterial similarity of 0.77. Conversely, adjacent cecal biopsies and cecal brushes had a median similarity of only 0.35 (0.27–0.47), while adjacent rectal samples had a similarity of 0.46 (0.44–0.49).

**Figure 4:**
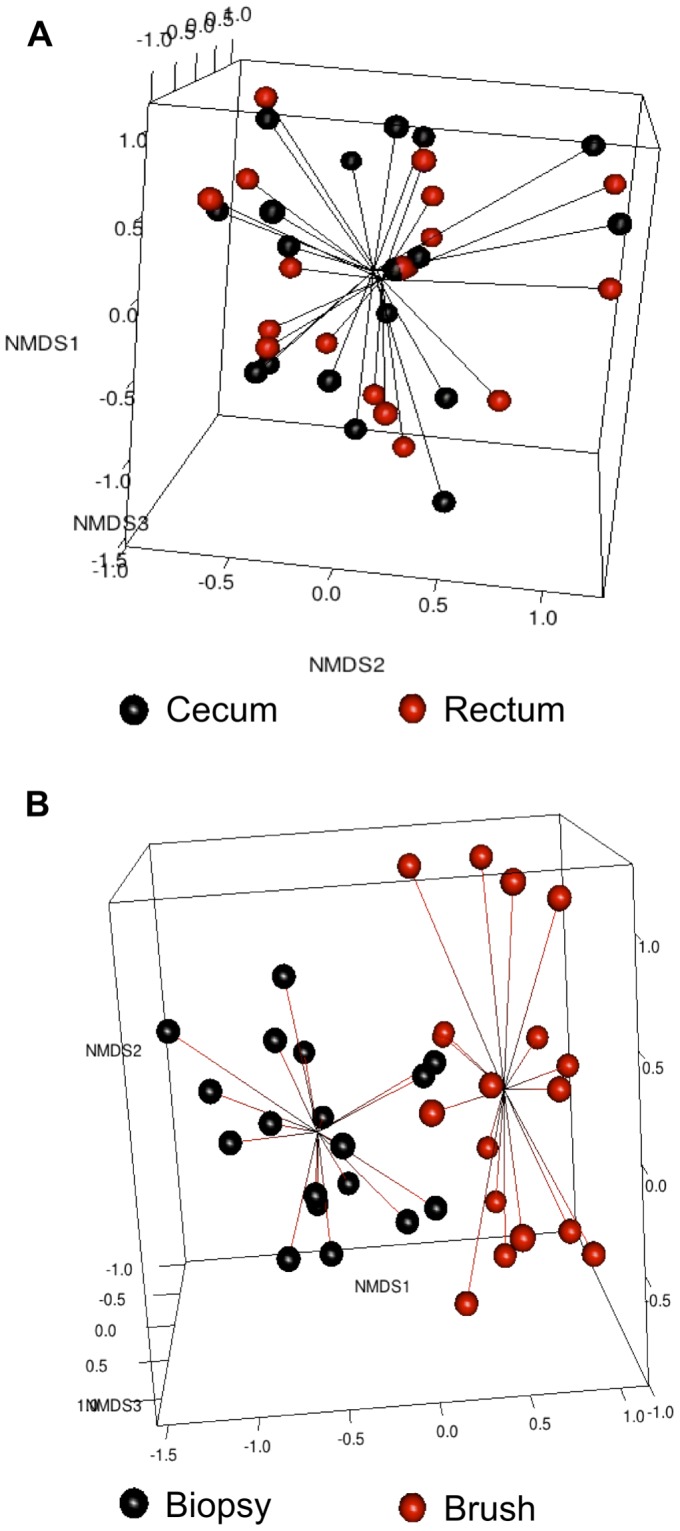
Separate clustering of luminal brush and mucosal biopsy samples between individuals. A. Non-metric Multidimensional Scaling (NMDS) reveals samples do not cluster based on colonic region (black = cecum; red = rectum). B. NMDS analysis reveals that samples cluster according to their cross-sectional location with respect to the host bacterial interface (black = Mucosal biopsy; red = luminal brush).

Three-dimensional visualization of a Bray-Curtis dissimilarity matrix by non-metric multidimensional scaling (NMDS) was performed on the combined cohort, assessing for relatedness between individuals, sample types and anatomic location (Figure 4). The pattern of clustering re-affirms that clustering occurred by sampling technique within individuals and not by colonic region. This was confirmed by Analysis of Similarity (ANOSIM), where there was no significant difference between cecal versus rectal samples (R = −0.043, *P* = 0.89), but a highly significant difference between sampling techniques (R = 0.47, *P* = 0.001) [41].

**Figure 5:**
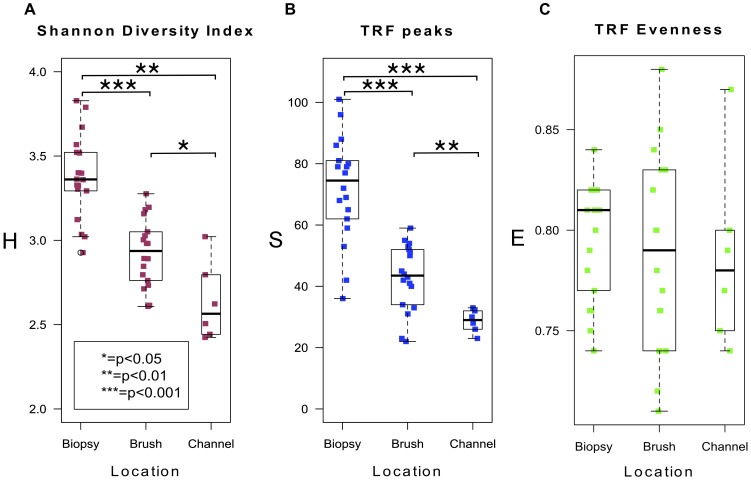
Reduced diversity in luminal brush samples when compared with mucosal biopsies. A. Boxplot of Shannon Diversity Index values for brush and biopsy samples (median value for biopsy samples 3.36 (3.3–3.5), median value for brush samples 2.94 (2.8–3.1) (*P*<.001). Median values for the working channel 2.6 (2.5–2.8). B. Boxplot of TRF abundance (median value for biopsy samples 75 (63–81), median value for brush samples 44 (36–52), (*P*<.001)). Median value for the working channel 29 (27–32). C. Boxplot of TRF evenness (median value for biopsy samples 0.81 (0.78–0.82), median value for brush samples 0.8 (0.75–0.83) (*P* = 0.591). Median values for the working channel 0.78 (0.76–0.8).

The Shannon Diversity Index (H′) was used to quantify biodiversity, based upon the number of species (calculated by using terminal restriction fragments as a proxy) and the evenness of their distribution (Figure 5A–5C). Species diversity was lower in brush compared with biopsy samples (3.36 (IQR 3.3–3.5) vs. 2.94 (2.8–3.1), *P*<.001)), as was the mean number of unique peaks per sample (75 (63–81) vs. 44 (36–52), *P*<.001). Evenness of distribution was however similar in both groups (0.8 (0.78–0.82) vs. 0.8 SD (0.75–0.83), *P* = .591).

**Figure 6:**
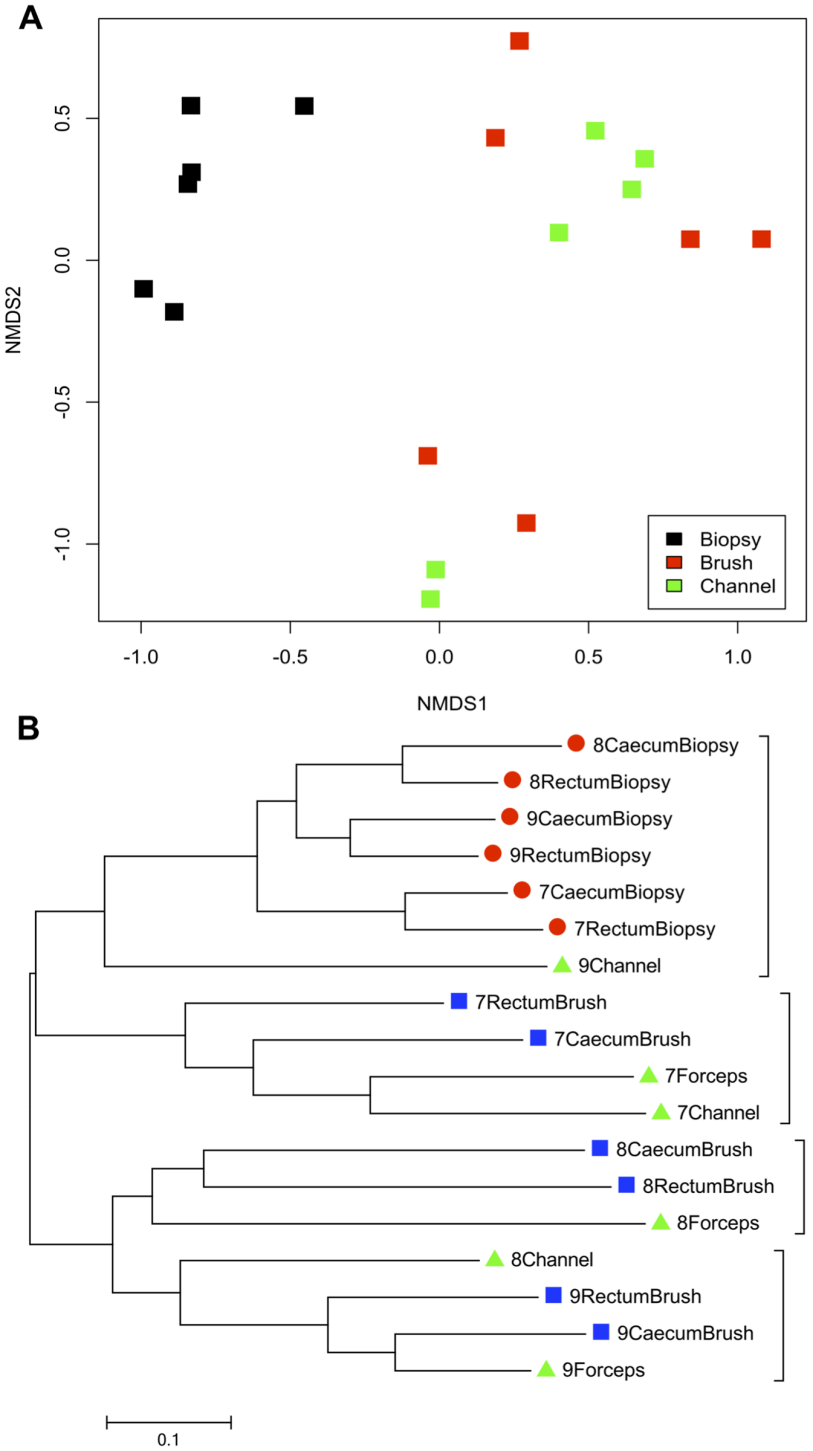
Poor discrimination between individuals by samples derived from colonoscope channel. A. NMDS plot (stress 0.085) highlighting separation of biopsy samples (black) from brush samples and samples taken from the working channel of the colonoscope (red and green, respectively). B. Neighbor-joining dendrogram of the same samples in A, illustrating that samples derived from the working channel do not discriminate as accurately between individuals as brush samples.

To confirm the lack of contamination of biopsy samples from fluid within the working channel, the outside of the biopsy forceps and the inside of the endoscope working channel were swabbed in 3 patients and a separate cluster analysis performed using NMDS and neighbor-joining methods. Samples from the working channel and the outside of the forceps clustered with luminal brush samples and quite distinctly from mucosal biopsies (Figure 6A). This finding re-iterates those previously published, that whole mucosal biopsies are uncontaminated within the jaws of the biopsy forceps [42]. However, samples derived from the working channel were less precisely able to discriminate between individuals (Figure 6B) and had lower diversity (median diversity 2.6 (2.5–2.8)).

## Discussion

In this study, community analysis of bacteria from the outside of the biopsy forceps and from within the working channel of the colonoscope illustrated that mucosal biopsies remain uniquely uncontaminated within the jaw forceps and that samples from the working channel are more similar to luminal samples than mucosa-associated communities. Furthermore samples from the working channel, which essentially represent directly aspirated fluid, are unreliable for loco-regional characterization and cluster less-reliably with brush samples from the same individual (Figure 6B).

The main aim of the study was to subsequently determine the major spatial variable, if any, which accounted for partitioning of microbial communities within the colon. The study was designed, *a priori*, to maximize separation by cluster analysis of the dominant variable (if one existed) by acquiring paired samples of adjacent luminal and mucosal communities with minimal distance between them in two regions that were maximally separated on the colonic longitudinal axis (cecum and rectum). Thus, if regional variability were dominant, the close approximation of luminal and mucosal samples would allow for this to become apparent with clustering, while if segregation of luminal and mucosal communities was the major determinant of biogeography, it would be evident despite the large distance in sampling from the cecum to the rectum (approximately 150–180 cm). T-RFLP analysis submitted to NMDS and neighbor-joining protocols illustrated that partitioning of the luminal and mucosal communities was evident, not just within individuals, but across the cohort as a whole and was statistically significant (Figure 4These findings are consistent with those that have been previously reported in studies of spatial microbial assessment, although with the addition of a paired brush sample demonstrating a partially distinct bacterial community [18], [20].

As inter-individual variability in the microbiota is so marked, it is perhaps surprising that this did not figure more prominently in the community analysis reported here[43]. Samples from the same individual by the same technique clustered together, however the separation of luminal and mucosal communities overcame this effect in the combined analysis. However, T-RFLP while ideally suited to the questions asked in the present study, is limited in its ability to resolve diversity at the species and strain level. T-RFLP fragments are likely to be redundant to a certain extent due to fragments of common length and sequence potentially existing between distinct species. The fact that fragment length is used as a proxy for species also imposes a certain limitation given that equally sized fragments are indistinguishable but could contain variable sequence reflecting generation from distinct species. As such, the statistically significant reduction in diversity described here in microbial brushes should be interpreted cautiously, as the sensitivity of T-RFLP may not resolve more closely related species-level phylotypes in these samples.

The effect of colonic lavage must also be acknowledged, for this can alter both the luminal [44] and mucosal microbiota [45]. With regard to the mucosal microbiota, there appears to be a reduction in phylotype richness and overall diversity following colonic lavage, however this does not appear to be directed at specific bacterial families nor does it seem to have more than a short-term effect on the microbiota [46].

Using microbial brush sampling, we have demonstrated that paired mucosal and brush samples are distinct with little variability across the long axis of the colon and a reduced diversity in brush samples. The exact niche that is represented by brush samples in the prepared colon is not entirely clear, however the consistently discrete community profile of brushes compared to their mucosal counterparts suggest they are dominated by luminal communities. Our study thus extends the findings of Araújo-Pérez et al. [22], who examined paired rectal swabs and biopsies, to include the entire colon and provides a method to access different colonic regions via a colonoscope.
